# Electronic coherence lifetimes of the Fenna–Matthews–Olson complex and light harvesting complex II†
†Electronic supplementary information (ESI) available: Sample growth and isolation, spectroscopic setup, simulated spectra used to verify subtraction procedure, additional figures. See DOI: 10.1039/c9sc03501j


**DOI:** 10.1039/c9sc03501j

**Published:** 2019-09-19

**Authors:** Shawn Irgen-Gioro, Karthik Gururangan, Rafael G. Saer, Robert E. Blankenship, Elad Harel

**Affiliations:** a Department of Chemistry , Northwestern University , 2145 Sheridan Rd. , Evanston IL 60208 , USA; b Department of Biology , Washington University in St. Louis , One Brookings Dr St. Louis , MO 63130 , USA; c Department of Chemistry , Michigan State University , East Lansing , Michigan 48824 , USA . Email: harelela@msu.edu

## Abstract

The study of coherence between excitonic states in naturally occurring photosynthetic systems offers tantalizing prospects of uncovering mechanisms of efficient energy transport. However, experimental evidence of functionally relevant coherences in wild-type proteins has been tentative, leading to uncertainty in their importance at physiological conditions. Here, we extract the electronic coherence lifetime and frequency using a signal subtraction procedure in two model pigment-protein-complexes (PPCs), light harvesting complex II (LH2) and the Fenna–Matthews–Olson complex (FMO), and find that the coherence lifetimes occur at the same timescale (<100 fs) as energy transport between states at the energy level difference equal to the coherence energy. The pigment monomer bacteriochlorophyll *a* (BChl*a*) shows no electronic coherences, supporting our methodology of removing long-lived vibrational coherences that have obfuscated previous assignments. This correlation of timescales and energy between coherences and energy transport reestablishes the time and energy scales that quantum processes may play a role in energy transport.

## Abstract 2

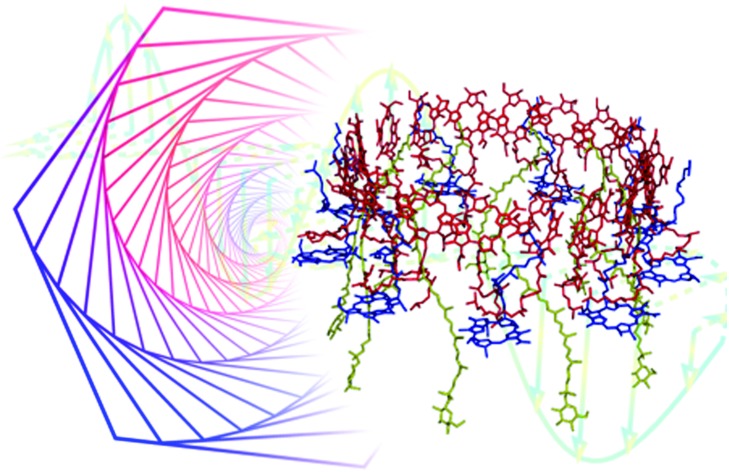
The study of coherence between excitonic states in naturally occurring photosynthetic systems offers tantalizing prospects for uncovering mechanisms of efficient energy transport.

## Introduction

The timescale in which a system maintains phase correlation, or coherence lifetime, dictates the observation of quantum interference at a macroscopic level.^1^ For electronic states, stochastic energy fluctuations cause coherences between the ground and the excited states to decay on the order of 10^–14^ seconds. In the late 2000's, the assumption that coherences between two excited electronic states should be on a comparable timescale was challenged when a series of experiments observed coherences for hundreds of femtoseconds, with possible functional implications for energy transport in photosynthetic systems.^2^–^8^ These initial experiments disputed the notions that the excited states' independent interaction with the thermal bath would rapidly destroy coherences between excitonic states (electronic coherences) and the conventional description of energy transfer using standard and modified Förster theory.^9^,^10^ However, more recent work has suggested that the spectroscopic signatures that began this debate, namely long-lived (hundreds of femtoseconds to picoseconds dephasing rate) coherences, can been attributed to originate from vibrations of the chromophores (vibrational coherences).^11^–^15^ In order to re-establish the timescale in which quantum effects may play a role in photosynthetic proteins, our study details a systematic method to extract electronic coherence lifetimes applied to two photosynthetic proteins, light harvesting complex 2 (LH2) and Fenna–Matthews–Olson (FMO) complex, using Multi-Dimensional Electronic Spectroscopy (*n*DES).

The *n*DES signal is generated with a series of three compressed broadband laser pulses, measured as a function of delay between the pulses, generating a 3D dataset. Two of these dimensions measure coherences between the ground and the excited state, which are popularly interpreted as the absorption frequency, *ω*_*τ*_, and the emission frequency, *ω*_*t*_. The “population time”, *T*, contains both population dynamics and the coherences between states within the excited or ground state manifold, which are the focus of this study and are detected as oscillations of the signal as a function of *T*. A recent theoretical study by Gelin *et al.* clearly partitions how electronic and vibrational degrees-of-freedom contribute to coherences at different timescales.^16^ At a <100 fs timescale, the time evolution is independent of nuclear degrees-of-freedom and is exclusively determined by electronic coupling. On a longer timescale, delocalized vibrations coupled electronic states form “vibronic” coherences, which eventually turn into purely vibrational coherences after a critical time *τ*_*D*_ ≅ 2π/*σ*, where *σ* is the dispersion of the static disorder. Understanding experimentally exactly when the signal transitions between different regimes of coherences gives microscopic details on the system-bath coupling. With a theoretical basis for what we expect to observe, our study aims to re-establish the timescale of electronic coherence in model photosynthetic proteins.

Previous studies have assigned large amplitude coherences that dephase at a ∼100 fs timescale to be electronic coherences.^15^,^17^–^19^,^20^–^22^ However, the multitude of overlapping signals at early timepoints makes it difficult to extract values of the frequency and dephasing rate of the electronic coherence. The most common method currently to extract these parameters is to try to extrapolate the frequency based off estimating the period of oscillation, but trying to approximate the frequency of a damped oscillator this way is fraught with errors, especially in the case spectral congestion. The experimental measurement of electronic coherence lifetime and frequency is further complicated because the time domain signals are convolved with the instrument response correlation time. It is known that laser power and phase fluctuations follow a 1/*f* distribution, leading to pulses at long time intervals to become increasingly uncorrelated.^20^ Thus, if a scan takes too long to acquire, coherence lifetimes will be artificially shortened. Previous measurements done by the corresponding author on LH2 showed coherences decaying with a ∼100 fs lifetime,^3^ but our current measurements see the same coherences out to many picoseconds as a result of the fast measurement, in which the entire scan is completed before significant loss of pulse correlation (typically, less than 2 s). Our study combines the rapid acquisition experimental methodology, which provides high SNR data, with a systematic procedure to extract electronic coherence to overcome these challenges.

## Results

**Fig. 1:**
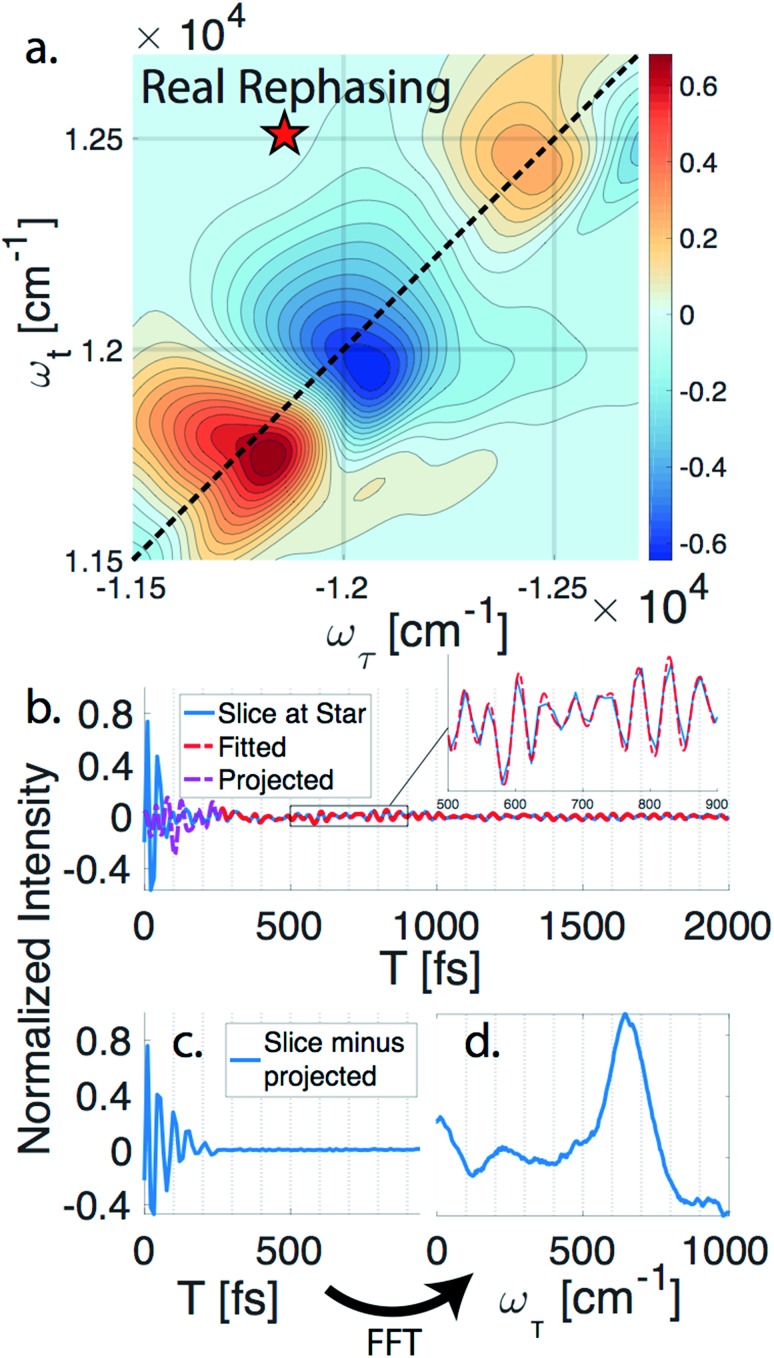
Methodology of extracting electronic coherence from the light harvesting complex II. (a) A slice of the real rephasing LH2 3DES spectrum is shown at *T* = 43 fs. (b) An example point from (a) is chosen at the star with population dynamics subtracted using global analysis. The residual (blue) is fit for later time points (red) and projected back to the early time points (purple). (c) The short lifetime coherences are revealed once the projected coherences are subtracted. (d) A Fourier transform is performed on (c) to reveal the line width and coherence frequency.

*n*DES spectra are taken for BChl*a*, LH2, and FMO, and in the case of LH2 and FMO a bimodal distribution of coherence lifetimes is observed. The long-lived coherences show agreement with a recent control experiment measured in our lab of vibrational coherences in BChl*a* and gives us confident assignment of long-lived coherences as primarily vibrational in origin.^13^ The contributions from population dynamics and vibrational coherences are removed with global analysis.^21^ Briefly, the 3D spectrum is written as a matrix multiplication of a basis set of real or complex exponentials and its amplitude for each point in the 2D spectra. Variable projection is used to find the basis set that best describes the full spectra, which essentially decomposes the spectrum into the contributions from each of the basis functions, allowing for reconstruction of spectra from a subset of components. This procedure is first run with a basis set of real exponentials, and the reconstructed spectra is subtracted to remove the contribution from population dynamics. The residual is run through global analysis again using a basis set of complex exponentials. In the second global analysis run, the early time points are excluded, and only later time points are considered. Thus, the reconstructed spectrum contains contributions only from the long-lifetime coherences. The resulting spectra is then subtracted from the population subtracted spectra to reveal the rapidly dephasing coherences in the typically congested first few hundred femtoseconds. This systematic method of subtracting vibrations is insensitive to the choice of cut-off time as long as one chooses a time longer than the electronic coherence lifetime because they have negligible contribution to the signal at long time points. We recently used a similar method to remove contributions to the signal from solute-only third order response and is described in more detail in this context in the methods section.^13^Fig. 1 diagrammatically shows how the vibration subtraction procedure is performed in LH2. The real rephasing spectra is shown in (a) at *T* = 43 fs. Once the population dynamics are removed, each point in the 2D spectrum contains oscillations as a function of *T*, which are the electronic and vibrational coherences (b). The long-lifetime coherences are fit at later time points and projected back to *T* = 0 with their respective amplitudes found through global analysis. Subtracting these contributions from long-lived coherences reports on the electronic coherences (c). These short-lived coherences can then be Fourier transformed to reveal the line width and coherence frequency (d).

**Fig. 2:**
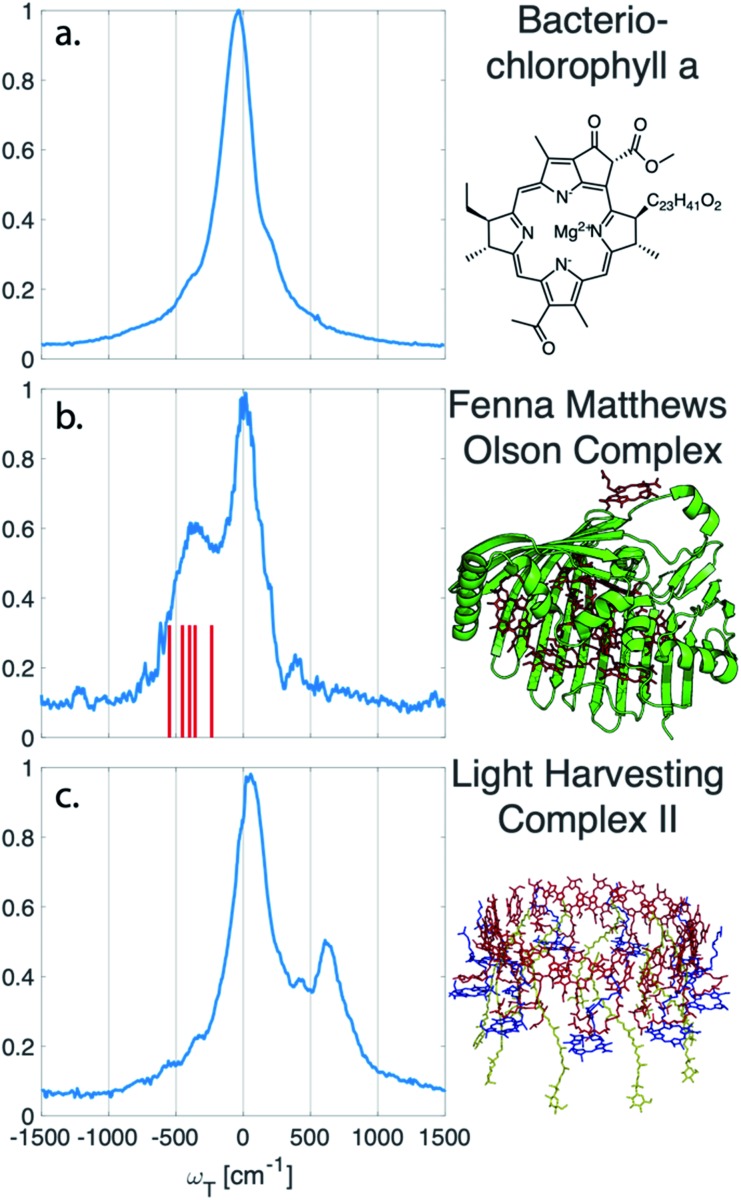
Sum of the electronic coherences over the entire 2D spectrum. (a) The BChl*a* spectra contains only vibrational coherences. With the long-lived coherences subtracted, no additional peaks are observed. Both (b) FMO and (c) LH2 have multiple electronic states within the bandwidth of the pulse and display fast decaying coherences. For FMO, excitonic energy level differences are drawn as a stick spectrum for differences between 330–550 cm^–1^. The closest excitonic energy level difference to the main peak is between excitons 7 and 2 at 400 cm^–1^, with the energy levels taken from ref. 22.

The vibrational subtraction analysis is first performed on BChl*a*, which is the monomer pigment found in both LH2 and FMO. Coherences of BChl*a* must be vibrational in origin since there is only one accessible excited electronic state within our pulse bandwidth. The sum of the Fourier transformed residual, like the one seen in Fig. 1(d), for the entire 2D spectrum can be seen in Fig. 2. This analysis on BChl*a* shows no remaining electronic coherence peaks, only a background that comes primarily from imperfect subtraction of real exponentials, non-exponential signals, and remaining 1/*f* noise.

Unlike BChl*a* where no electronic coherences are possible, LH2 and FMO may show both electronic and vibrational coherences owing to the high-density of excitonic states within the pulse bandwidth. The same subtraction procedure applied on LH2 and FMO, as seen in Fig. 2(b and c), show broad peaks. For FMO, the main peak is centred around ∼350 cm^–1^. FMO is a system of 8 BChl, with one weakly surface bound BChl likely to become disassociated during the process of isolation and purification. The seven remaining excitonic states have energies ranging from 12 120 to 12 670 cm^–1^ and the energy level difference of some of the excitons are displayed as sticks in Fig. 2(b).^22^ The closest excitonic energy level difference to the observed peak is between excitons 7 and 2 using the numbering of BChls chosen by Fenna and Matthews.^23^ It is thought that excitons are delocalized across 1/2, 3/4, and 5/6/7, and even though excitons 7 and 2 are neighbouring chromophores, they are predicted to have relatively weak excitonic coupling.^24^ Previous reports of rapidly dephasing coherences in FMO found that the lowest oscillation frequency was well above the distribution of excitonic energy level differences (∼600 cm^–1^), not matching any exciton energy differences.^15^ In LH2, the electronic coherence extracted is centred at ∼630 cm^–1^. However, since the excitonic energies are ambiguous, no proposed energy level differences are drawn. The main complicating factor is the high density of excitonic states with similar energies that can be modulated by specific instances of disorder.^25^ From the absorption spectrum, the energy level difference of the two BChl*a* rings, B800 and B850, is 735 cm^–1^. Previous work on a mutant of LH2 with the B800 band removed observed an electronic coherence at 687 cm^–1^, which was assigned to be between B850 and a higher lying excitonic state B850*.^17^ Although there is uncertainty to which states the observed coherences are between, it is clear that both FMO and LH2 contain rapidly dephasing coherences that do not exist in BChl*a*.

Global analysis with a complex exponential basis retrieves the lifetime and energy of the electronic coherence peaks. For LH2 the main peak is centred at 680 cm^–1^ with a lifetime of 81 fs, and for FMO it is at 390 cm^–1^ with a lifetime of 57 fs. However, we find that global analysis sometimes has difficulty in retrieving lifetimes of broadened peaks and is sensitive to the number of exponentials used to fit a given peak. Thus, the lifetimes extracted with global analysis are supplemented with the 1/*e* decay rates from the time domain of individual points in the 2D spectra. Estimating the electronic decoherence rates from the time traces, like the one seen in Fig. 1(c), gives ∼80 and ∼60 fs dephasing rates for the largest peaks of LH2 and FMO, respectively. These dephasing rates can be compared to the expected dephasing rates, which are revealed using the antidiagonal slice of the 2D spectrum. The antidiagonal slice reveals the homogenous broadening of a state, which is related to the dephasing time between the ground and excited state by *τ* = [π*cΔ*]^–1^, where *Δ* is the FWHM of a fitted Lorentzian. This holds true even in the presence of strong vibronic coupling.^26^ While FMO's expected dephasing rate matches the coherence lifetime, the estimated lifetimes of the B800 and B850 states in LH2 gives 56 and 42 fs respectively (slices and fits shown in ESI†). The measured electronic coherence lifetime of ∼80 fs is unexpectedly longer than the dephasing rate of the individual states. Gelin *et al.* offers another method to predict the coherence lifetime using the expression 
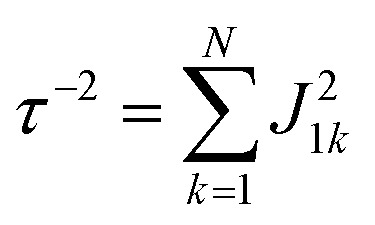
, where *N* is the number of chromophores and *J* is the inter-site electronic coupling strength.^16^ Comparison to their predicted value of 53 fs of the electronic coherence lifetime of FMO, we find good agreement. However for LH2, which has more chromophores and higher coupling strength (at least in the B850 band), we should expect the coherence lifetime to be even shorter.^32^ This trend holds true for the rates extracted from the anti-diagonal slices, but not for the electronic coherence measured in the time domain. Using both metrics, we find that the measured electronic coherence lifetime is longer than predicted. The electronic coherence frequency and lifetimes obtained from our methodology are summarized in Table 1.

**Table 1:** Comparison of electronic coherence lifetime and frequency extracted through global and Fourier analysis

Protein	Global Lifetime	Global Frequency	Fourier Lifetime	Fourier Frequency	Expected Dephasing
FMO^*a*^	57 fs	390 cm^–1^	60 fs	350 cm^–1^	60 fs
LH2^*b*^ 17	81 fs	680 cm^–1^	80 fs	630 cm^–1^	42–56 fs

^*a*^Previous work saw coherences on ∼100 fs timescale but did not extract rates or energies.

^*b*^Previous work on mutated LH2 with the B800 band removed found electronic coherences at 680 cm^–1^ with a lifetime of 137 fs.

**Fig. 3:**
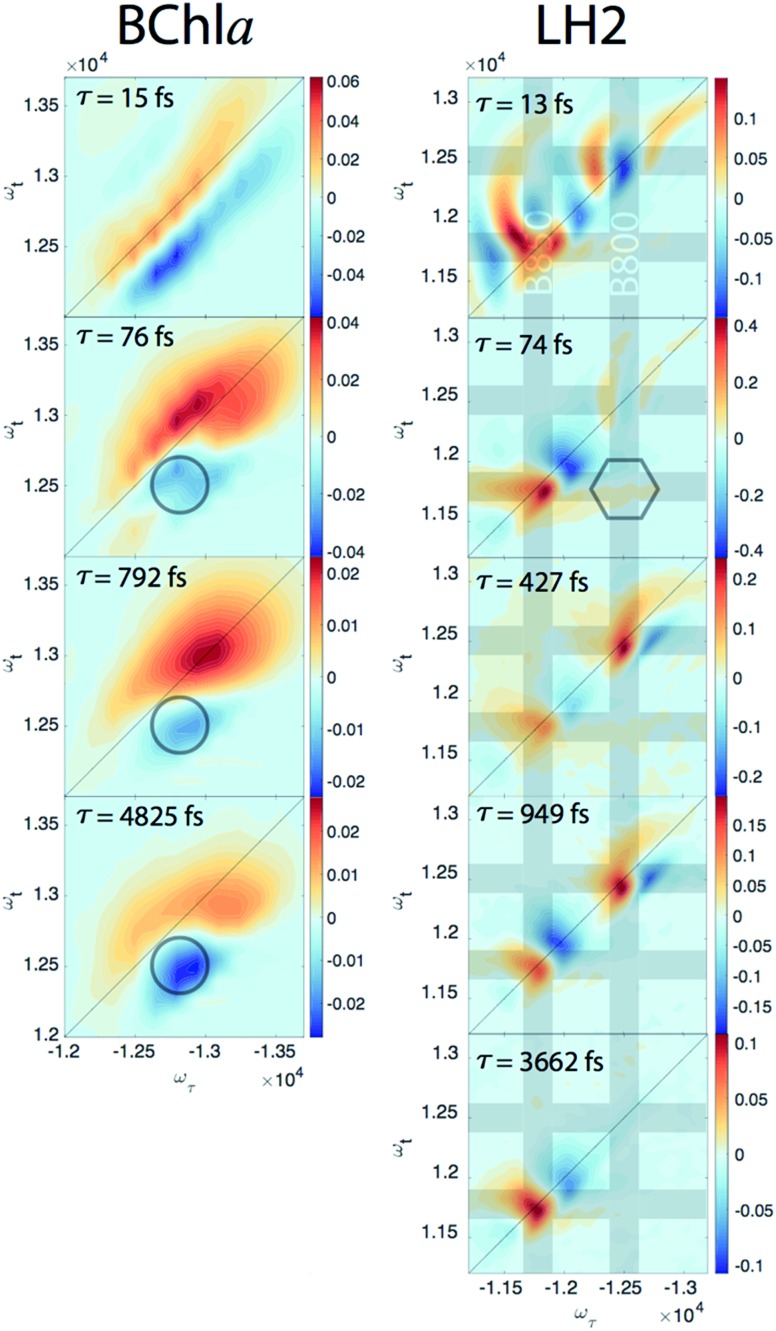
DAS beatmaps of BChl*a* (left) and LH2 (right). The BChl*a* assignments are aided by only having one electronic state within the bandwidth of the laser and simpler dynamic processes. The BChl*a* 13 fs component indicates a global shift energy and is attributed to a Stokes shift convolved with instrument response. The 76 fs component appears to be an internal relaxation process with signal growth at the low energy side of the spectra (highlighted in the circle). The 74 fs LH2 component most likely has similar internal relaxation processes, but also appears to have amplitude at the downhill cross-peak [*ω*_*τ*_, *ω*_*τ*_] = [–12 400, 11 700] cm^–1^, located in the hexagon, at roughly the electronic coherence energy. Grey bands are located on the energies of the B800 and B850 states.

Since our experiment establishes that the electronic coherences are happening at a <100 fs timescale, for coherences to be functional and not simply a phenomenon universal to all molecules there has to be biologically relevant processes happening on the same timescale. For FMO, the kinetic rates between all sites has been well characterized and shows that this timescale is irrelevant for exciton transport.^22^ However, in LH2, <100 fs timescale processes have been implicated in both energy transfer between the rings through intermediate states and population transfer to states with mixed excitonic and charge transfer character process.^27^–^29^ Thus, we will take a closer look at the population dynamics extracted during the first iteration of global analysis. This viewed the signal as a sum of contributions from real exponentials, e^–*t*/*τ*^, described with a single parameter, which is the decay lifetime *τ*. The associated amplitude for a given exponential decay at each point on the 2D spectra is called the Decay Associated Spectra (DAS) beatmap and shows all species that have dynamic components over *T* with a given rate. The difficulties of interpreting DAS in general are explored in the ESI,† but interpretation of the BChl*a* spectra is simplified because it only contains one electronic excited state with simpler dynamical processes compared to FMO or LH2. Furthermore, the BChl*a* 2D spectrum contains no contributions from ESA, so positive signal on the DAS beatmap indicates a decay of signal while negative areas indicate signal growth. In Fig. 3, it can be seen that the DAS for BChl*a* and LH2 both possess similar fast (<100 fs) components. In the BChl*a* 15 fs component, the signal grows in on the Stokes shifted side of the diagonal while decaying on the anti-Stokes side, indicating a global shift of energies. However, since this component is on the timescale of our pulse duration, we assign this to instrument response. The other components of the BChl*a* indicate growth of signal pumping at higher energies and probing at lower energies, [*ω*_*τ*_, *ω*_*τ*_] = [–12 800, 12 500] cm^–1^, indicated on Fig. 3 with circles. This growth of signal at the lowest energy state suggests these components are internal relaxation processes. Utilizing the BChl*a* assignments to inform the DAS beatmaps in LH2, the 15 fs component is similarly assigned to be instrument response. However, for the LH2 74 fs component, non-internal relaxation processes distinct from the BChl*a* 76 fs DAS beatmap appear. Amplitude at the downhill cross-peak at [*ω*_*τ*_, *ω*_*t*_] = [–12 400, 11 700] cm^–1^, highlighted on Fig. 3 in the hexagon, indicating a pump and probe energy level difference of ∼700 cm^–1^, shows energy transfer at the same timescale and energy as the electronic coherence. Dynamic processes that may be occurring at a <∼100 fs timescale are discussed later, but it is possible that the same state in coherence are experiencing energy transfer at this ultrafast timescale. The slower rates of LH2 are attributed to decay of the ground state bleach of the B800 and B850 rings and population transfer between the two rings. Specifically, the ∼3.6 ps decay shows up only on the B850 band of LH2 and the broad bi-excitonic ESA feature. The similar lifetimes, also seen in pump-probe experiments, have been used to justify that the ESA originates from the excited B850 ring.^30^ The other two slower 472 and 949 fs components are difficult to assign due to the similarity of features. It is possible that they are the same component, which would agree with previous reports have indicated that the B800 and B850 transfer rate occurs with *τ* = 700 fs.^31^

## Discussion

Identifying the lifetime and energy of electronic coherences limits the role of electronic coherences to the <100 fs timescale and in turn what biological functions may be implicated. Although it is possible that electronic coherence coincidentally occurs at the same timescale as dynamic processes between states of the same energy level difference, the large body of work on LH2 provides clues to which states may be involved in the electronic coherence. One likely possibility is that the coherence observed here is between the B850 and B850* state, which would be the same coherence previously observed in the mutated species by the Engel group.^17^ B850* refers to the higher lying excited states of the B850 ring's excitonic progression. Although the typically named *k* = ±1 transition contains most of the total oscillator strength, symmetry breaking allows for other transitions that overlap the B800 band. This model predicts that energy transport within the B850 band occurs on a <100 fs timescale.^29^ Atomistic modelling of LH2 has shown that in the unmutated species, the B850* state mixes with the B800 state and that excitation becomes delocalized across both rings. This mixed state is thought to be an intermediate between the B800 and B850 ring, mediating stepwise downhill transport.^28^ Thus, coherences within the excitonic manifold of B850 offer explanations to both the <100 fs electronic coherence and energy transfer. However, another mutant study where the B850 band is shifted to 810 nm revealed a dark state with charge transfer character around 850 nm,^27^ but with no electronic coherences reported. This study revealed that excitations in the B800 band are transferred to the CT state in a <100 timescale. In the unmutated species, this CT state is theorized to mix with the B850 ring further adding energy levels to an already congested energy landscape. Due to the multitude of possible states involved, we cannot confidently assign the states in coherence purely based off the energy of the electronic coherence observed.

## Methods

### Global analysis

The 3D spectrum, *Y*, is a *N*_*τ*_ × *N*_*T*_ × *N*_*t*_ matrix. This can be rewritten as a matrix multiplication problem, *Y* = *GT*, where *G* is a *N*_*τ*_ × *N*_*t*_ × *m* matrix and *T* is a *m* × *N*_*T*_ matrix, and *m* is the number of basis sets. *T* is what is referred to as the basis set, as each 1 × *N*_*T*_ row of the matrix is either a real or complex exponential depending on if populations or coherences are fit. *G* is the associated amplitude of each basis. In the case of real exponentials, the *N*_*τ*_ × *N*_*t*_ × 1 matrix associated with a given exponential is the DAS beatmap. Since *G* can be solved for by multiplying the original data by a basis set, *G* = *YT*^–1^, the entire optimization can be boiled down to finding the basis set that best describes the data. Using variable projection, this minimizes the number of parameters needed to be solved. To fit later times, only data past a certain time point is considered so that the basis set now is described by a *m* × (*N*_*T*_ – *N*_exclude_) matrix, where *N*_exclude_ is the number of early time points no longer considered. To extrapolate, the *G* from the late times is multiplied by a new basis set that now includes the early time points, keeping the same coefficients for each complex exponential basis. Our methodology of fitting later time points and projecting the fit back to the early time points is tested out on simulated spectra in the ESI.†

### Sample isolation

LH2 and BChl*a* are isolated from *Rhodobacter sphaeroides* 2.4.1. FMO is extracted from *Chlorobaculum tepidum*. Details can be found in the ESI.†

## Conclusions

Observation of excitonic coherences in photosynthetic complexes on the timescale of biologically relevant dynamics constrains the time and energy scales that electronic coherences may play a role. Electronic decoherence lifetimes were extracted from FMO and LH2, while similar analysis on their pigment monomer, BChl*a* revealed no early time coherences. While the majority of exciton transport in these systems happens on a picosecond timescale and is dominated by spatially directed dipole coupling, our study suggests that there may still be a niche function for electronic coherence at a <100 fs timescale.

## Conflicts of interest

There are no conflicts to declare.

## Supplementary Material

Supplementary information
