# Supplementary material for: Electronic coherence lifetimes of the Fenna–Matthews–Olson complex and light harvesting complex II
Source: Chem Sci. 2019 Sep 19;10(45):10503–9. doi: 10.1039/c9sc03501j (PMC7003877; doi:10.1039/c9sc03501j)
Supplement: Supplementary file 1 [file SC-010-C9SC03501J-s001.pdf]

Electronic Supplementary Material (ESI) for Chemical Science.
This journal is © The Royal Society of Chemistry 2019

Electronic Coherence Lifetimes of the Fenna-Matthews-Olson Complex and Light Harvesting Complex II

Supporting information

Shawn Irgen-Gioro[a], Karthik Gururangan[a], Rafael Saer[b], Robert Blankenship[b], Elad Harel[a]

[a] Department of Chemistry, Northwestern University, 2145 Sheridan Rd. Evanston IL 60208

[b] Department of Biology, Washington University in St. Louis, One Brookings Dr. St. Louis, MO 63130

Corresponding Author

elharel@northwestern.edu

Experimental Methods

Sample Growth and Isolation

Rhodobacter sphaeroides 2.4.1 (ATCC 17023™) is grown semi-anaerobically under lamps at 30°C in a shaker in ATCC Medium 550: R 8 A H medium. Cells were pelleted with a Sorvall RC6+ ultracentrifuge and resuspended in a 20 mM Tris buffer (pH 8) in preparation to be lysed. The concentrated cell solution is first disrupted with a sonicator for 5 mins at a 30% duty cycle and then run through a French press five times to ensure complete breakage of the cellular membrane. The resulting suspension is then centrifuged again to collect the large membrane fragments into a pellet. The pellet is again diluted with the same Tris buffer now containing 1% lauryldimethylamine oxide (LDAO) by weight to the desired OD for our measurement and shaken for an hour at room temperature. The LDAO helps solubilize the protein and reduces the amount of dynamic scatter. The solution is once again centrifuged to reduce remaining large membrane fragments and run through an AKTAxpress FPLC to ensure purity. The resulting proteins are concentrated using Amicon centrifugal filters so the optical density of is 0.3 OD passing through a 200 um flow cell from Starna. The purified protein is passed through a 100 nm syringe filter to allow for smooth passage in the 220 nm filter used in the closed loop peristaltic pump system.

BChl*a* is extracted from the same cell pellet the LH2 is extracted from. The BChl*a* is solubilized in methanol (plus .005 M sodium ascorbate) and stirred in the dark and cold for about half an hour. This mixture is then sedimented by centrifugation and repeated until the sedimentation is colorless. The resulting supernatant contains both BChl*a* and carotenoids, which is separated through AKTAxpress FPLC to ensure purity. The concentration is increased by rotovaping to remove excess methanol to achieve a suitable optical density for spectroscopic measurements.

The FMO complex was isolated from *Chlorobaculum tepidum* cells cultured in CL medium (DOI: 10.1128/AEM.67.6.2538-2544.2001) according to the procedure in Saer et al. (DOI: 10.1016/j.bbabio.2016.04.007) with some modifications. Briefly, cells were harvested by centrifugation at 7000 x g, and resuspended in 20 mM Tris HCl, 2 mM MgCl2, pH 8.0. The cell suspension was sonicate to lyse the cells. This lysate was centrifuged at 12,000 x g for 10 minutes to remove cell debris, and subsequently ultracentifuged at 100,000 x g for 1 hour to pellet the cell membranes. Membranes were resuspended in 20 mM Tris HCl, pH 8.0, and sodium carbonate was added to a final concentration of 0.4 M. The mixture was left to stir overnight at 4 ^{o}C . The alkalinity of this mixture was neutralized by adding sodium bicarbonate to a concentration of 0.4 M, and the mixture was then ultracentrifuged for 2 hours at 235,000 x g. The supernatant was dialyzed overnight against 20 mM CAPS, pH 10.5 and subsequently purified with a strong anion exchanger. FMO eluted from the column at 250 mM NaCL. The eluate was concentrated with a 50,000 kDa ultrafilter and further purified by gel filtration chromatography. Pure FMO protein exhibited a 260:371 nm absorption ratio of under 0.6.

The concentrations of all the samples were adjusted so that when flowed through a .2 mm cell, an OD of .3 was achieved for the absorption maximum. All experiments were conducted at room temperature.

Data Collection

The broadband coverage of the B800 and B850 was achieved using a homebuilt second harmonic non-collinear optical parametric amplifier (2H-NOPA) pumped with a PHAROS fundamental pulse running at 200 kHz and compressed with chirped mirrors (Layertech 109347).

The signal is collected in the GRAPES geometry and the phase of the signal is retrieved using the phase of a non-resonant solvent scan, which is purely dispersive. The resulting phased spectra is then checked with a degenerate pump-probe experiment taken with the same sample and setup with one of the beams blocked. This transient-absorption measurement is equivalent to our 3DES signal summed along the excitation dimension and has the added benefit of already being properly phased relative to the probe pulse. The same phase mask is applied to our entire signal. The ability to not have to phase the data for every time delay is due to passively phase stabilizing the interferometer as well as collecting the entire 3DES dataset in under 2 seconds, getting rid of most of the 1/f phase noise.

The ability to collect the data quickly also gets rid of much of the 1/f amplitude noise that hinder measurement sensitivity. Typical “point-by-point” measurements of 3DES require two time delays to be sampled. Our experiment focuses the beams to a line instead of a point, which allow us a spatial dimension in which we can encode a time delay. Pulses 1 and 2 are tilted relative to each other so that each row has a slightly different time delay between them. Pulse 3 is parallel to 2, so that time delay is scanned with a stage. The Newport XPS stage system we use can output triggers at preset locations without stopping. We use this feature to “rapid-scan” the delay between 2 and 3, using the stage to trigger the camera to take a picture at a given time delay. Not stopping the stage and running an Andor Xyla 5.5 high speed CMOS camera at 650 hz allows us to complete at 128x916x2560 pixel scan in under 2 seconds. A more detailed explanation can be found in previous work.

Simulation of 3D Electronic Spectra

Simulated 3D ES were created to both test the accuracy of the zero-quantum coherence subtraction procedure and identify factors that cause asymmetry in the 4WM beating spectra. The simulation calculates the optical response of a system of N chromophores and M vibrational modes described by the following Hamiltonian (neglecting anharmonicity):

$$\hat{H} = \hat{H}_{el} + \hat{H}_{vib} + \hat{H}_{el-el}$$

$$= \sum_{n=1}^{N} \epsilon_n |n\rangle\langle n| + \sum_{m=1}^{M} \hbar\omega_m \hat{b}_m^\dagger \hat{b}_m + \sum_{n=1}^{N} |n\rangle\langle n| \sum_{m=1}^{M} S_{nm}(\hat{b}_m^\dagger + \hat{b}_m) + \sum_{n,n'=1}^{N} J_{nn'}(|n\rangle\langle n'| + |n'\rangle\langle n|)$$

where $|n\rangle$ and $\hat{b}_m$ denote the electronic site basis and vibrational annihilation operator. S_{nm} is the Huang-Rhys factor coupling the mth vibrational mode to the nth chromophore (related to the dimensionless displacement γ_{nm} via $S_{nm} = \frac{\gamma_{nm}^2}{2}$) and $J_{nn'}$ is the interchromophore Coulomb

coupling. The Hamiltonian was diagonalized and the eigenvalues were obtained to calculate the 3rd-order optical response functions in the impulsive limit (pulse duration goes to 0) and under the rotating wave approximation using a sum-over-states procedure for the photon echo pathway ($k_{sig} = -k_1 + k_2 - k_3$). A pulse bandwidth of 1500 cm^{-1} is used to window the observable signals. Using the Condon approximation to separate electronic and nuclear contributions to the dipole transition moment, the response functions can be calculated in the Fourier domain using the following expressions:

$$\begin{cases} R^{\mathrm{ESE}}(\omega_1,\omega_2,\omega_3) = |\mathbf{d}|^4 \sum_{\alpha a \beta b} \langle a|\alpha\rangle\langle b|\alpha\rangle\langle \beta|a\rangle\langle \beta|b\rangle I_{a\alpha}(\omega_1) I_{ab}(\omega_2) I_{\beta b}(\omega_3)\rho_{\alpha\alpha}(0) \\ R^{\mathrm{GSB}}(\omega_1,\omega_2,\omega_3) = |\mathbf{d}|^4 \sum_{\alpha a \beta b} \langle a|\alpha\rangle\langle b|\alpha\rangle\langle \beta|a\rangle\langle \beta|b\rangle I_{a\alpha}(\omega_1) I_{\alpha\beta}(\omega_2) I_{\beta b}(\omega_3)\rho_{\alpha\alpha}(0) \\ R^{\mathrm{ESA}}(\omega_1,\omega_2,\omega_3) = |\mathbf{d}|^4 \sum_{\alpha a b f} \langle a|\alpha\rangle\langle b|\alpha\rangle\langle f|a\rangle\langle b|f\rangle I_{a\alpha}(\omega_1) I_{ab}(\omega_2) I_{f b}(\omega_3)\rho_{\alpha\alpha}(0) \end{cases}.$$

In these expressions, Greek letters denote states in the ground-state manifold while Roman letters are states in the excited state manifolds (including f-states). The nuclear overlaps are denoted by expressions of the form $\langle a|\alpha\rangle$ while the electronic dipole moment $|d|$ is assumed to be the same for all interactions. The lineshape $I_{ab}(\omega_i)$ represents either a Gaussian, Lorentzian, or Voigt function centered at frequency $\omega_{ab} = \omega_a - \omega_b$ with FWHM broadening of Γ_{ab} along frequency dimension ω_i. Note that the Voigt functions used in this work had equal homogeneous and inhomogeneous broadenings of Γ_{ab}. The total signal was calculated as the sum of the signals from the three different pathways. The laser pulse shape was not incorporated into these simulations.

Vibrational Coherence Subtraction Procedure

To test the vibrational coherence subtraction procedure's ability to isolate the electronic coherences in the system, a simple three-level system (two excited state sharing a common ground state) was simulated with electronic dephasing time of 100 fs and vibrational dephasing of 3.3 ps, as shown in Figure S1(a). All three signals were simulated but notice that for such a system, only the GSB and ESE will produce signals within the observable bandwidth; the f-states are well out of reach. One vibration at 215 cm^{-1} is present in the system with a Huang-Rhys factor of 0.14 for both excited state manifolds. For this model, Lorentzian lineshapes were used to parameterize the spectrum along all 3 coherent dimensions. The spectrum for T > 1.33 ps was fit using global analysis to extract the long-lived vibrational coherences whose beat maps and coherent components are shown in Figure S1(c)-(d). The dynamic components were extrapolated back to T = 0, recombined with their beat maps and subtracted from the total 3D spectrum using the same procedure described in the main text. Figure S1(e) shows the population time dynamics of the spectrum for each step of this procedure; the blue curve is the original time evolution and the purple shows the late-time fit. This procedure fits the late times and propagates to 0 with good accuracy. Their residual is shown in red, isolating the features of the electronic coherences. The Fourier transform of the residual in Figure S1(f) shows broad peaks centered at near 800 cm^{-1}. The black lines are placed at exactly ± 800 cm^{-1}; it is evident that some error is introduced in identifying the excitonic coherence frequencies using this procedure, but the offset is minimal.

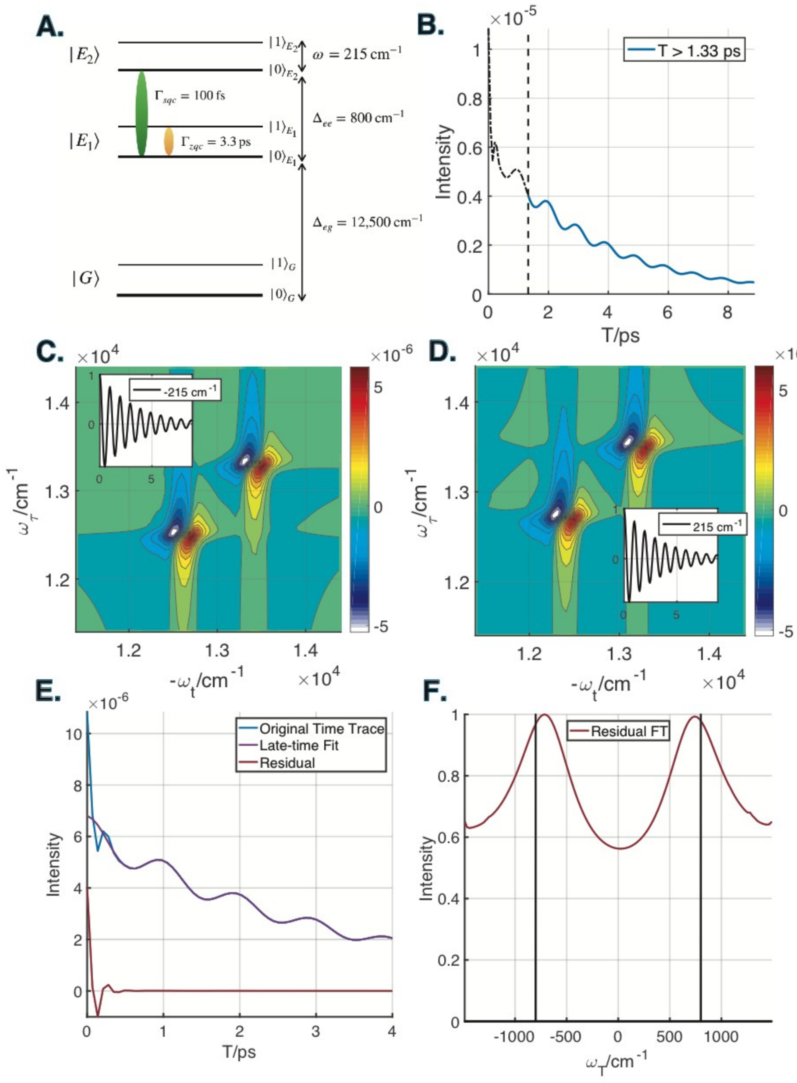

Figure S1. (a) Energy level diagram of the three-level system used to illustrate the vibrational coherence subtraction procedure. Single-quantum (electronic) coherences dephase at 100 fs while zero-quantum (vibrational) coherences diphase at 3.3 ps. (b) Waiting time dynamics of simulated spectrum with 1.33 ps marked so show where the spectrum was fit for the late-time regime. (c)-(d) Zero-quantum coherence beat maps corresponding to the vibrational coherence at 215 cm-1 (shown in the inset). (e) Time traces of each step of the subtraction procedure. (f) Fourier transform of the residual demonstrating that the procedure can find the position and broadening of the electronic coherence.

ESA-induced Beating Spectrum Asymmetry

Upon examining the equations for the response functions, one finds that the waiting time coherences for the ESE and ESA must be symmetric over positive and negative values since the frequencies ω_{ab} and ω_{ba} will be sampled evenly. The GSB will be symmetric for all energies up the highest thermally-occupied state (indicated by the α-states) for the same reason, but all of its other frequencies will only appear on the negative side. Since the total signal is the sum of these three pathways, asymmetry can be introduced in myriad ways. Since the GSB and ESA always nearly overlap, the asymmetry caused by the GSB simply manifests as negative coherence frequencies having larger amplitude than the corresponding positive ones. This is a somewhat trivial source of asymmetry. The ESA on the other hand can appear at any point on the spectrum based on the location of the f-states which tend to densely span a large range of energy in multichromophoric systems. Furthermore, since the ESA is a negative signal, it can produce highly asymmetric features based on partial cancellation due to its position relative to the GSB and ESE. To investigate the ESA-induced asymmetry, the spectrum of a 4-level system with 1 ground state, 2 singly-excited states, and 1 f-state was simulated and the relative amplitude of positive and negative peaks in the beating spectrum was plotted as a function of f-state position, as defined in Figure S2(a). Only the ESE and ESA were simulated to investigate the effect of ESA-induced asymmetry. For a "balanced" configuration in which the energy gap ω_{f1} equals the 5500 cm^{-1} (the sum of the Δ_{eg}= 5000 cm^{-1} and Δ_{ee} = 500 cm^{-1}), the ESA feature appears exactly on the off-diagonal positions coinciding with the ESE signal. Since these signals have the same dipole moments, they completely cancel. On the other end, if we shift the f-state down by 500 cm^{-1} to 5000 cm^{-1}, the positive peak cancels as in Figure X(c), and if it is shifted up to 6000 cm^{-1}, the negative peak cancels as shown in Figure S2(d). Figure S2(e) shows the continuous trend of partial cancellation with f-state position.

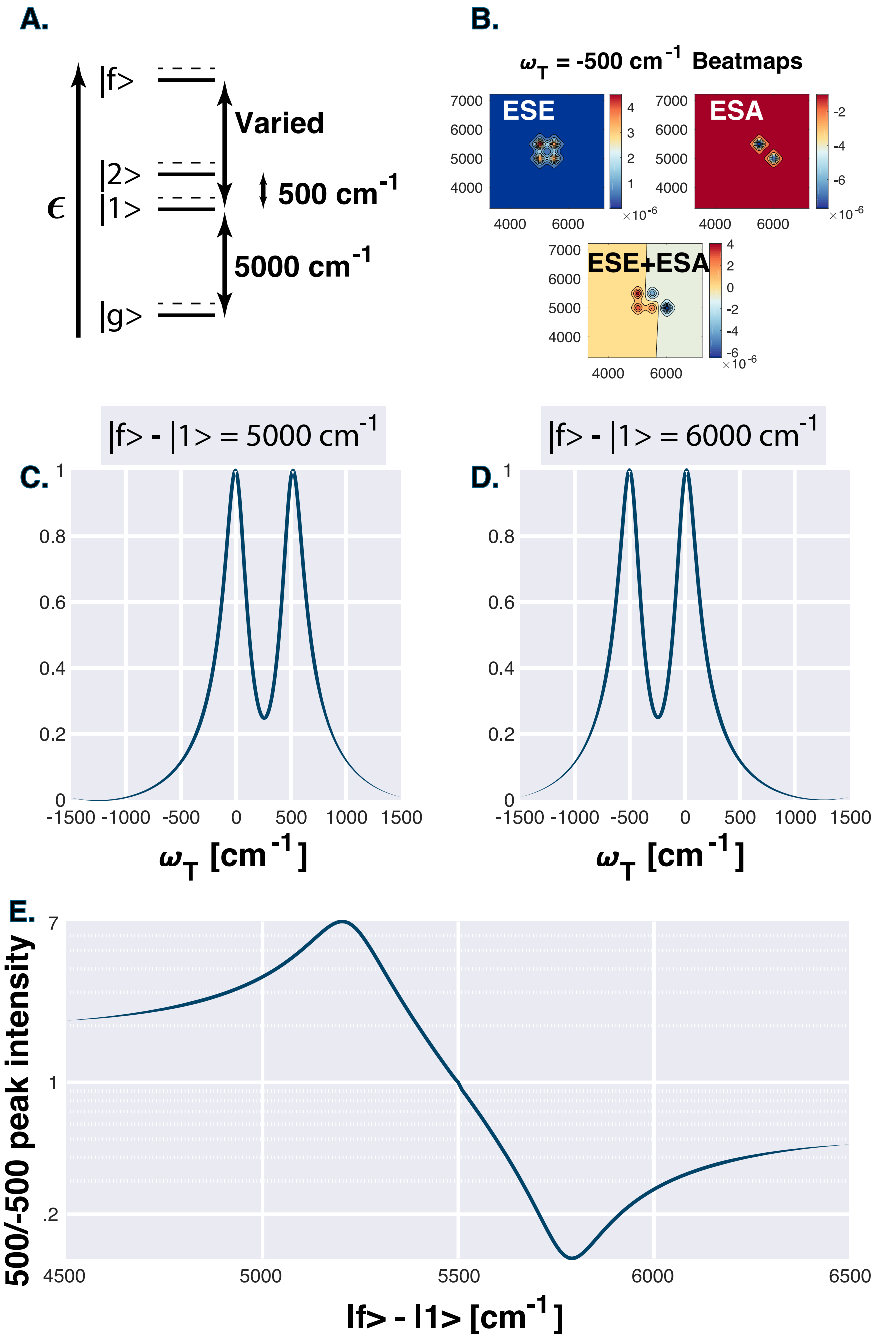

Figure S2. (a) Energy level diagram for the 4-level system used to explore ESA-induced asymmetry. (b) Images of the spectrum along the electronic dimensions showing the canonical structure of the ESE signal and the ESA signal at off-diagonal positions. The ESA can be shifted left to right by tuning the f-state gap. (c) Beating spectrum of the total signal for an f-state at 5000 cm-1, cancelling out the negative peak. (d) Beating spectrum of the total signal for an f-state at 5500 cm-1, cancelling out the positive peak. (e) Relative amplitude of positive and negative SQC peak as a function of f-state position.

Subtraction of Vibrational Signals in FMO and BChla

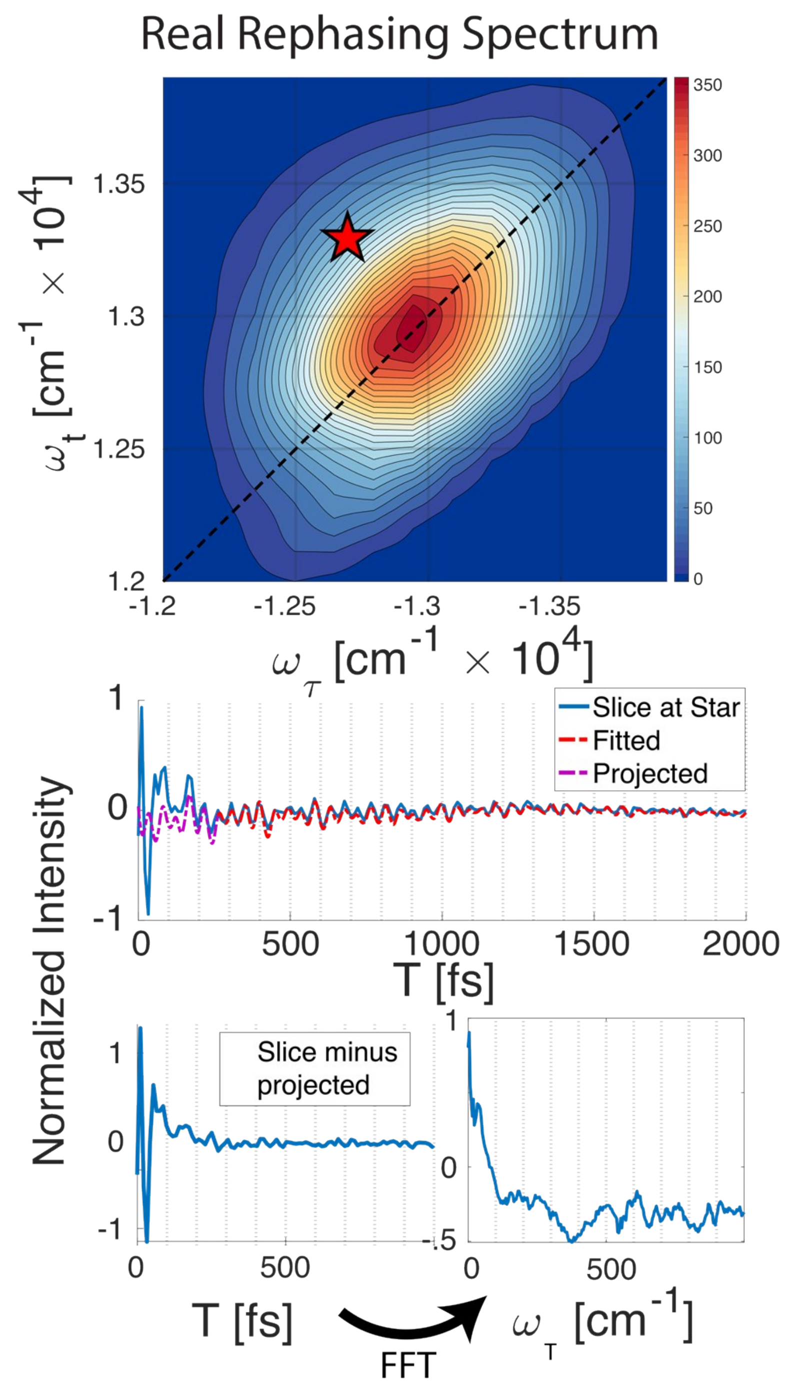

Figure S3. The 2D-SQC plot of BChl*a* is all positive with no ESA. The back propagation method leaves no peaks in the frequency domain.

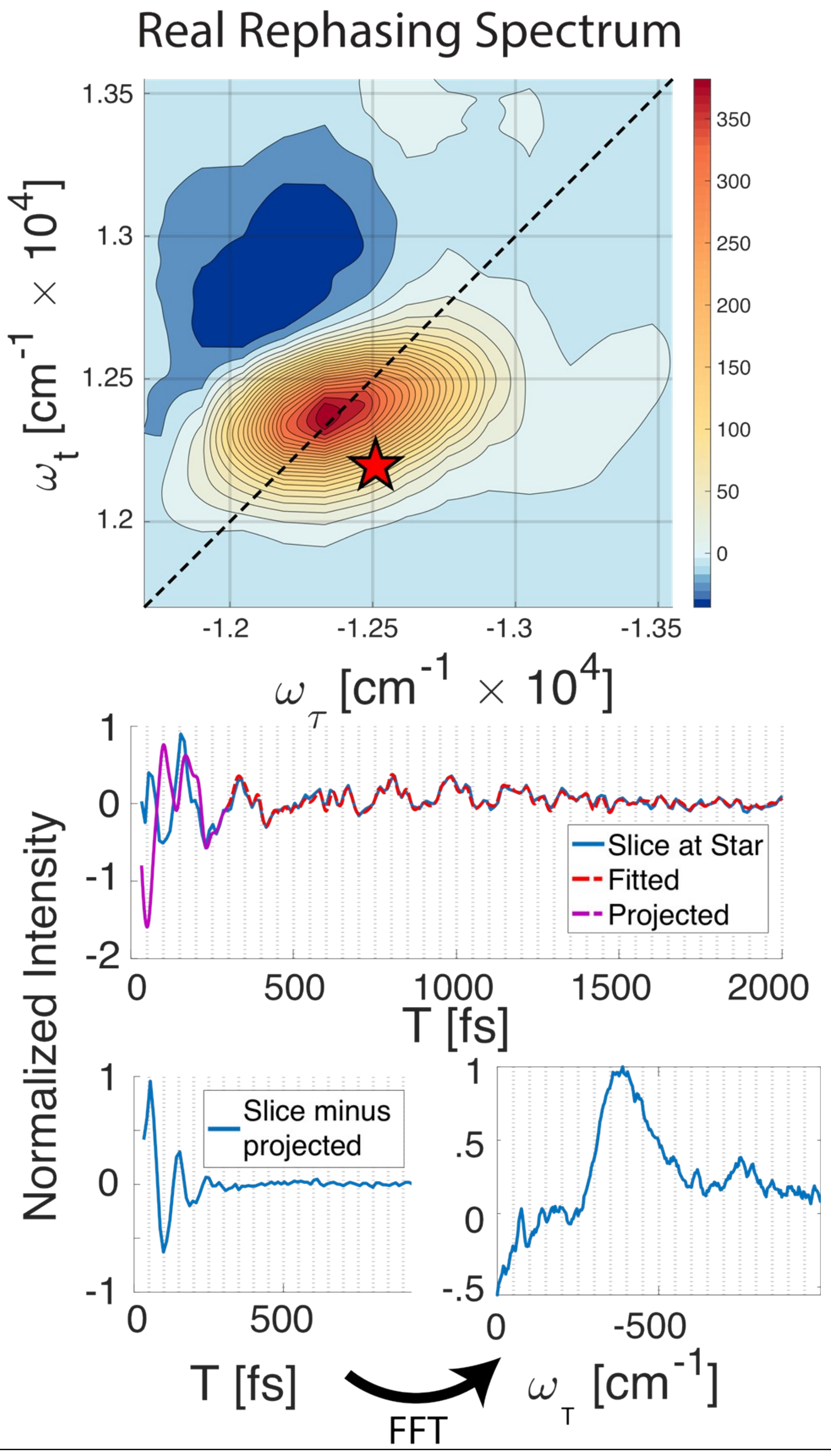

Figure S4. FMO 2D-SQC plot at T = 250 fs and example subtraction slice. Estimating the dephasing from the FWHM of the FT of a single slice gives a dephasing time of ~60 fs using $\tau = [\pi c \Delta]^{-1}$

DAS Interpretation

Although it is tempting to associate growth and decay of signal from different parts of the DAS as transfer between the respective states, the real picture is more nuanced. When as DAS regression is performed, the time evolution of the 3D signal along population time is fit to a linear combination of exponentials

$$I^{DAS}(\omega_\tau,\omega_t,T) = \sum_k c_k(\omega_\tau,\omega_t)e^{-|\lambda_k|T}.$$

If we assume population transfer is described by a kinetic rate model, the real dynamic evolution is parameterized by a rate matrix **K** where element K_{ij} controls the rate of excitation transfer from state $|i\rangle \to |j\rangle$. The dynamic evolution is governed by

$$I(\omega_\tau,\omega_t,T) = \sum_{ij} \tilde{c}_{ij}(\omega_\tau,\omega_t)e^{K_{ij}T}$$

where $\tilde{c}_{ij}(\omega_\tau,\omega_t)$ is a beat map that characterizes the dynamic process transferring excitation from $|i\rangle \to |j\rangle$. In transient absorption spectroscopy, these are often called species-associated spectra (SAS). These two representations are related by diagonalizing $K = V\Lambda V^{-1}$:

$$I(\omega_\tau,\omega_t,T) = \sum_{ij} \tilde{c}_{ij}(\omega_\tau,\omega_t)e^{K_{ij}T} = \sum_{ijk} \tilde{c}_{ij}(\omega_\tau,\omega_t)V_{ik}V^{-1}_{kj}e^{\lambda_k T}.$$

Therefore, the DAS fit recovers the eigenvalues of the population rate matrix **K**; the corresponding DAS beat maps are linear combinations of the SAS beat maps. This is illustrated in for the kinetic scheme shown in figure S5(a). The DAS components and beat maps indeed recover the eigenvalues, as shown in figure S5(b). Furthermore, it is clear that while there are only 4 beat maps, there are 16 individual transfer processes (elements in **K**), meaning that each DAS map contains mixed information from multiple transfer processes. This is the inherent difficulty in interpreting the DAS; however, when the peaks are well-separated, the DAS is very useful for determining which states are related by kinetic processes. These considerations do throw ambiguity into interpreting DAS, but the time scales in which processes occur still offer a clue to the dynamics happening in each DAS. Though the rate of transfer is not definitive, the fact that cross peaks do show up between different states (as in the 74 fs DAS of LH2) indicate they are not independent states in the <100 fs regime. Lastly, it is worthwhile to point out that the eigenvalues of triangular matrixes are the entries on the main diagonal. This means in the limit of low uphill energy transfer, the **K** matrix becomes a lower triangular matrix and the extracted rates are the main diagonal entries.

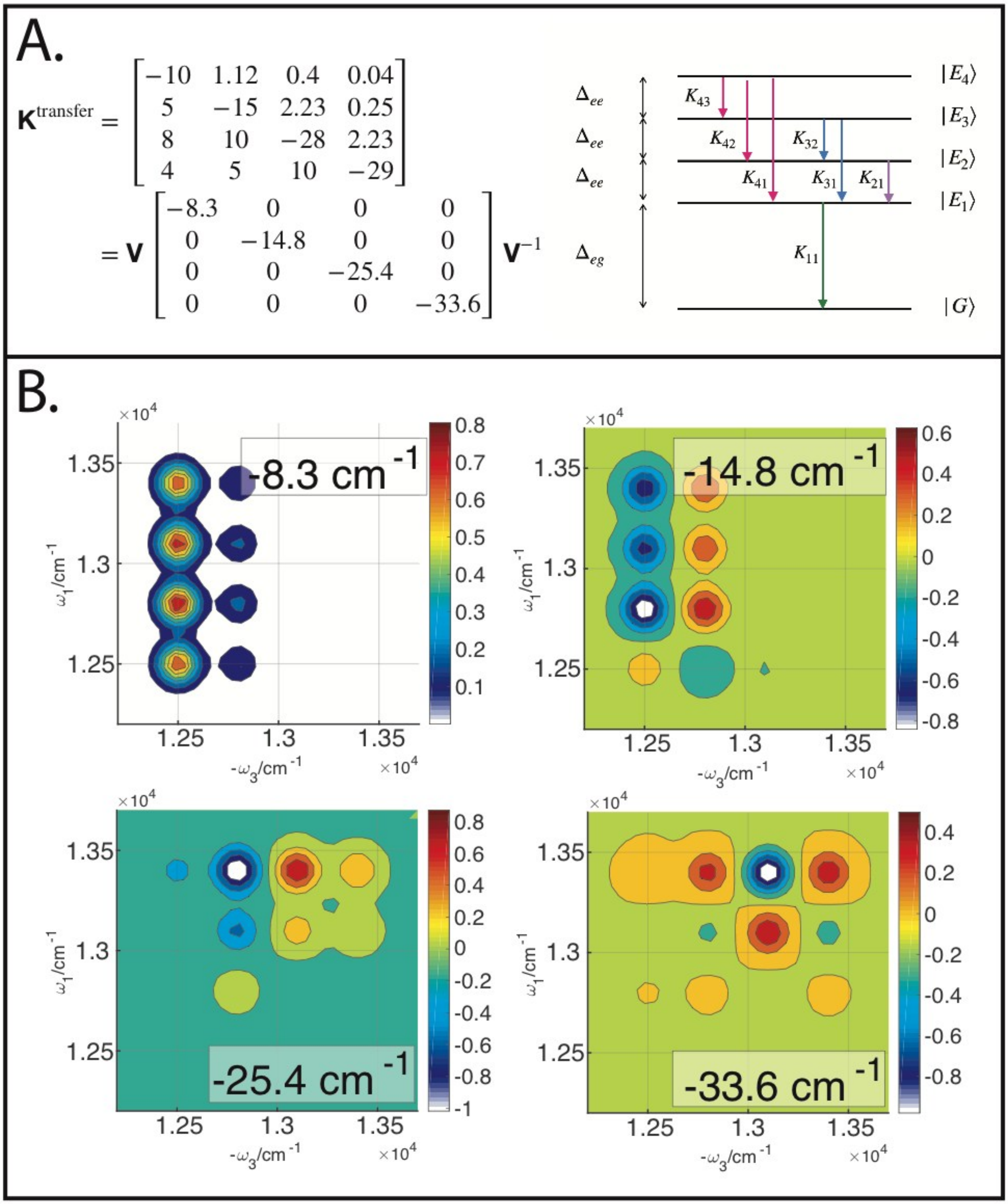

Figure S5. Simulation of DAS which highlight the difficulty in assigning a rate constant between two states. (A) The kinetic scheme used in the simulation. A four level system with transfer rates between states described in the full kinetic matrix **K**. (B) DAS extracted from **K** matrix, showing that the DAS extracts only the eigenvalues of the full **K** matrix.

FMO DAS

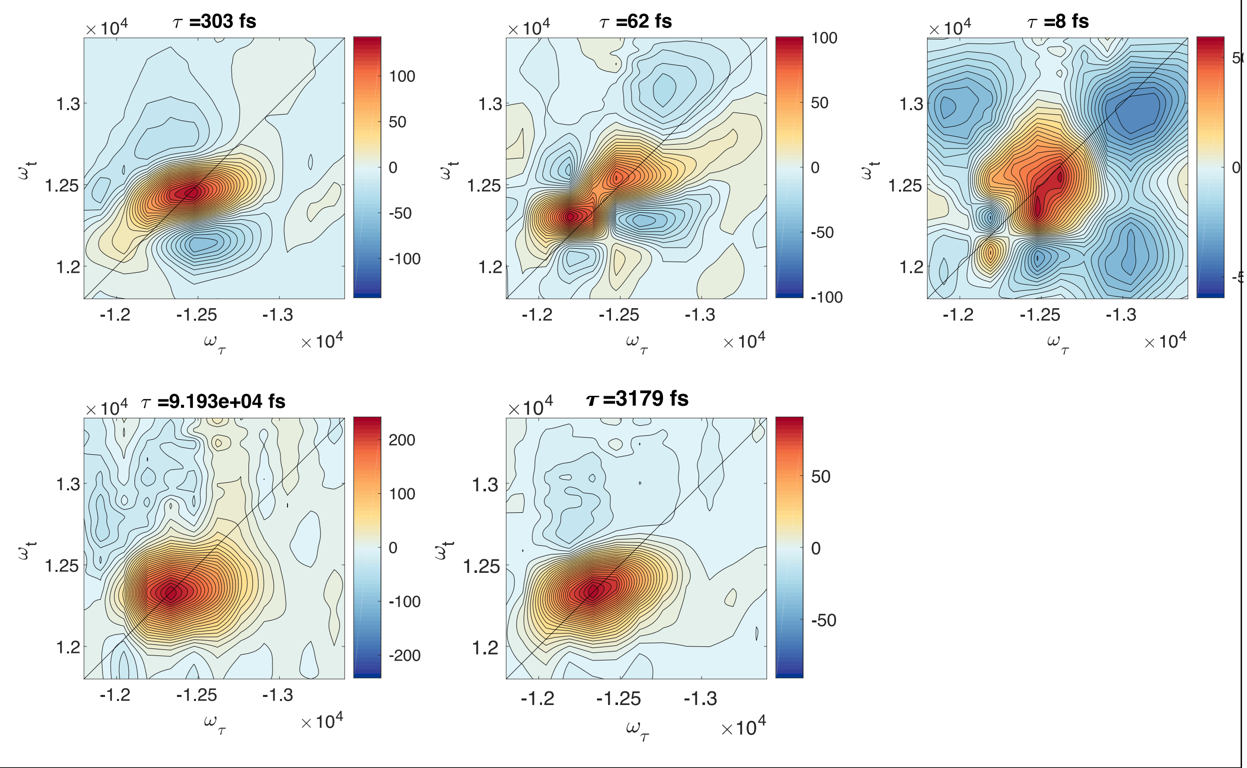

Figure S6. DAS of the FMO complex.

Antidiagonal Slices of LH2

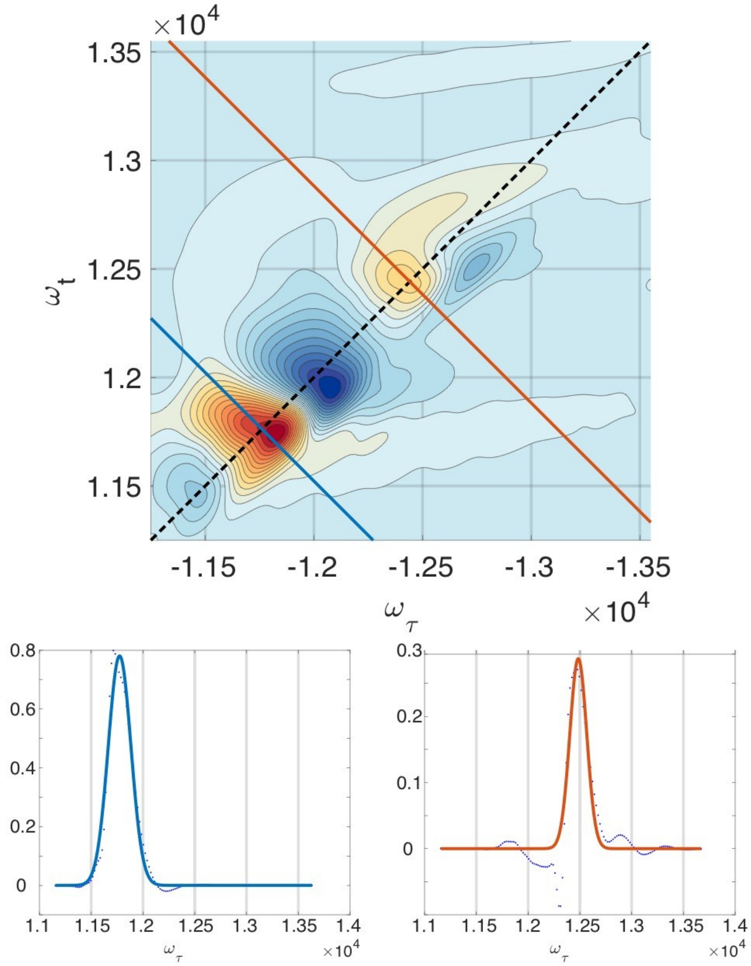

Figure S7. The dephasing lifetimes of the B800 and B850 bands are estimated from the anti-diagonal slices of the 2D-SQC at 30 fs. The dephasing lifetime is calculated using $\tau = [\pi c \Delta]^{-1}$ is found to have a shorter lifetime than the electronic dephasing of the system.

DAS of Non-Resonant Scan

The instrument response is captured by taking a scan of the buffer solution. The DAS for this scan is shown in figure S8. The 10 fs decay component is the decay of the non-resonant signal, while the long time component is simply the DC offset and the remaining non-exponential components of the signal. The 6 fs component represents what chirp remains in our pulse. The negative signal is a growth of signal, meaning that the pulse overlap is different for different colors. Note that differences in the growth and decay of the non-resonant signal are along the diagonal and not along the anti-diagonal.

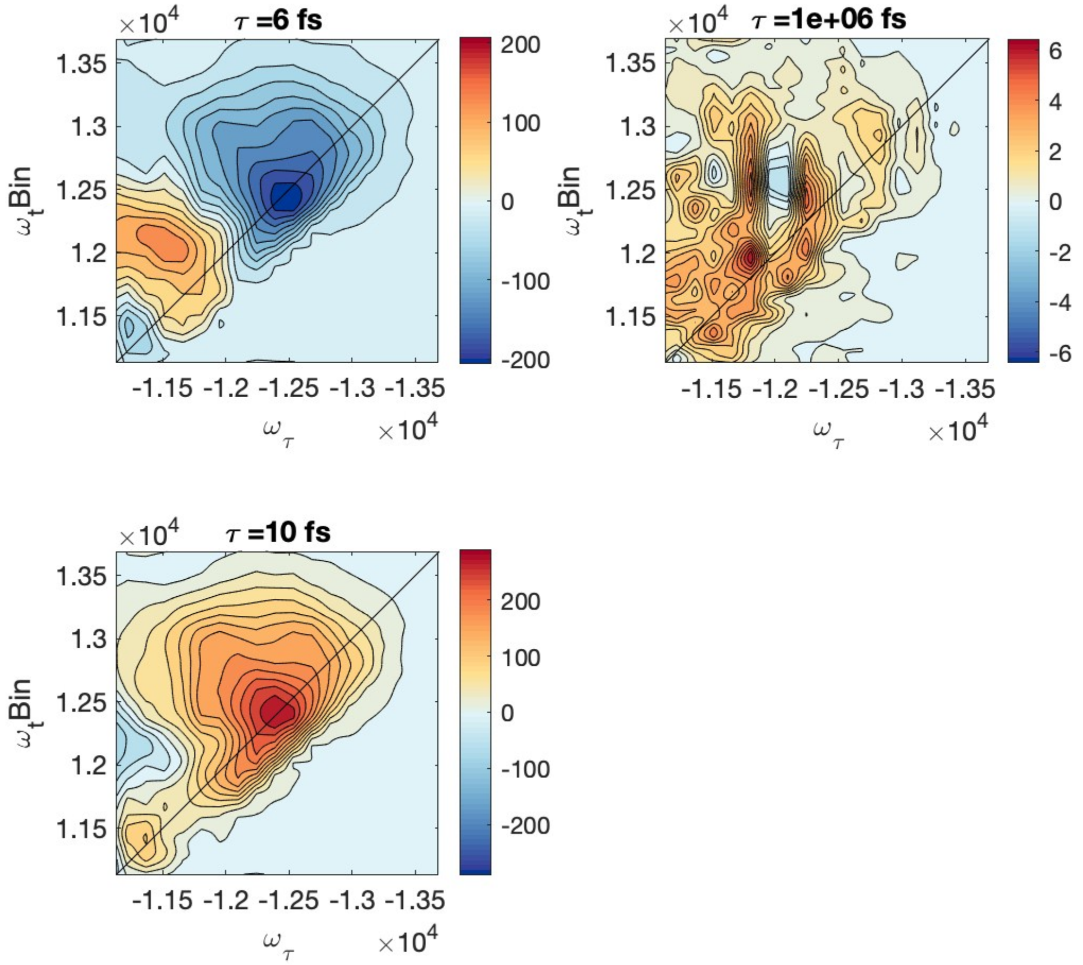

Figure S8. DAS of non-resonant scan of buffer.
